# Heterogeneous Coupling between Interdependent Lattices Promotes the Cooperation in the Prisoner’s Dilemma Game

**DOI:** 10.1371/journal.pone.0129542

**Published:** 2015-06-23

**Authors:** Cheng-Yi Xia, Xiao-Kun Meng, Zhen Wang

**Affiliations:** 1 Tianjin Key Laboratory of Intelligence Computing and Novel Software Technology, Tianjin University of Technology, Tianjin 300384, China; 2 Key Laboratory of Computer Vision and System (Ministry of Education), Tianjin University of Technology, Tianjin 300384, China; 3 Interdisciplinary Graduate School of Engineering Sciences, Kyushu University, Fukuoka, 816-8580, Japan; Wenzhou University, CHINA

## Abstract

In the research realm of game theory, interdependent networks have extended the content of spatial reciprocity, which needs the suitable coupling between networks. However, thus far, the vast majority of existing works just assume that the coupling strength between networks is symmetric. This hypothesis, to some extent, seems inconsistent with the ubiquitous observation of heterogeneity. Here, we study how the heterogeneous coupling strength, which characterizes the interdependency of utility between corresponding players of both networks, affects the evolution of cooperation in the prisoner’s dilemma game with two types of coupling schemes (symmetric and asymmetric ones). Compared with the traditional case, we show that heterogeneous coupling greatly promotes the collective cooperation. The symmetric scheme seems much better than the asymmetric case. Moreover, the role of varying amplitude of coupling strength is also studied on these two interdependent ways. Current findings are helpful for us to understand the evolution of cooperation within many real-world systems, in particular for the interconnected and interrelated systems.

## Introduction

Cooperation behavior is ubiquitous among social individuals ranging from micro-organisms to animals and human beings [1, 2], which, however, seems inconsistent with the prediction of Darwinian theory [3]. To resolve this issue, evolutionary game theory has become one of key paradigms behind many scientific disciplines [4, 5]. Borrowing from the analysis methods of statistical physics [6] and network science [7–11], network reciprocity is amongst the most well-known mechanisms that may sustain cooperation in evolutionary games [12]. That is to say, when players are arranged on the spatially structured topology and interact only with their direct neighbors, cooperation can survive by means of forming compact clusters, which minimizes the exploitation and protect those cooperative individuals located within the interior of such clusters [13]. After this seminal discovery, the role of spatial topology and its various underlying promotion mechanisms in evolutionary games have been intensively explored [14–17]. Typical examples include complex networks [18–22], the presence of mobile agents [23–26], the diversity between players [27–31], reward and punishment [32–37], co-evolutionary scenarios [38–44], to name but a few.

In spite of great accumulated progress, huge quantities of existing works simply assume that players have only interactions with others in the same network, which is actually inconsistent with the empirical observations. In reality, individuals are simultaneously the elements of more than one network in most, yet not all, natural and engineering systems [45, 46], and it thus becomes instructive going beyond the traditional isolated network theory and proposing a novel framework. The interdependent networks, defined as the combination class of interrelated networks in a nontrivial way, recently become a fundamental tool to quantitatively describe the interaction among networks as well as between these constituents [47]. This architecture is actually suggested in the research of network robustness, where even seemingly irrelevant changes in one network can have catastrophic and very much unexpected consequence in another network [48–50]. Along this way, interdependent network has become a hot topic of general interest, is extensively employed to study diverse subjects upon them, such as disease spreading and immunization [51–53], random walk [54, 55], traffic [56, 57], voting dynamics [58, 59] as well as the emergency and promotion of cooperation [60, 61].

With regard to game models on interdependent networks, the dynamic process between different networks are mainly coupled via utility function and strategy information transition [62]. In the pioneering work, Jin et al. proposed a fashion of symmetric utility: individual utility is composed of its own payoff and that of its counterpart from another network, which is controlled by coupling strength [63]. It is interestingly unveiled that cooperation will simultaneously be promoted below a critical threshold, but above which the phenomenon of symmetry breaking (namely, cooperation level is unequal again) takes place, which is induced by asynchronous expansion between heterogeneous strategy couples of both networks. Resorting to importance of synchronization across networks, another recent report [64] shows that simultaneous formation of correlated cooperator clusters on interdependent networks can enhance cooperation to a completely dominated level, which enriches the context of traditional network reciprocity. Besides, the utility function is further extended to biased coupling way [65], partially inter-correlated fashion as well as co-evolution between coupling strength and imitation ability [66]. While for information coupling, it can be assumed that individual decision depends on not only the payoff advantage of a neighbor but also how popular the adapting strategy in the network of its counterpart [67]. Furthermore, Attila and Perc also displayed that the excess correlation of players between two networks can promote the cooperation to an extremely high level [68]. Meanwhile, Jiang and Perc investigated the impact of external links between networks on the cooperation behavior, in which external links lie between a lattice and complex network (e.g., random or scale-free network), and the results revealed that an intermediate interdependence optimally facilitates the spreading of cooperative behaviour between groups [69].

If we look back upon the above-mentioned literatures, however, we can find a common characteristic: the coupling strength between networks is always homogeneous, irrespective of coupling fashion. While in the realistic life, heterogeneity plays a significant role in human behavior, even including the individual decision-making process [70]. An interesting question thus takes place: if there exists the heterogeneous coupling strength within the framework of interdependent networks, how does it affect the spatial reciprocity of cooperation? Aiming to answer this question, here we consider the heterogeneous coupling in interdependent networked games. It is unambiguously unveiled that increasing heterogeneity is beneficial for the dominance of cooperation yet may impede cooperation in some special conditions.

## Methods and Models

We consider an evolutionary prisoner’s dilemma game that is characterized with the temptation to defect *T* = *b* (the highest payoff received by a defector if playing against a cooperator), reward for mutual cooperation *R* = 1, and the punishment for mutual defection *P* as well as the sucker’s payoff *S* (the lowest payoff received by a cooperator if playing against a defector) equaling 0. As a standard practice, 1 < *b* ≤ 2 ensures a proper payoff ranking (*T* > *R* > *P* ≥ *S*) and captures the essential social dilemma between individual and common interests [13]. The players are staged on two square lattices, each of size *L* × *L*, where initially each player *x* is designated either as a cooperator (*s*_*x*_ = *C*) or defector (*s*_*x*_ = *D*) with the equal probability. Based on direct interactions with nearest neighbors, player *x* can obtain its accumulated payoff *π*_*x*_. Due to the interdependence between networks, individual utilities used to determine fitness are determined by itself and its parter *x*′ from another network (*i.e*., via external links between corresponding players), in the mode *U*_*x*_ = *π*_*x*_+*α* × *π*_*x*′_. The parameter 0 ≤ *α* ≤ 1 represents the coupling strength (or the strength of external links), and consequentially, the larger its value, the higher the potential increase of utility of two players that are connected by the external link.

It is worth mentioning that the interdependence of two networks does not allow strategies to be transferred across networks. The game is thus iterated forward in accordance with the Monte Carlo simulation procedure comprising the following elementary steps. First, a randomly selected player *x* acquires its utility *U*_*x*_ by playing the game with its nearest neighbors and taking into account also the potential addition to the utility stemming from the possible external link. Next, one randomly chosen neighbor *y* also acquires its utility *U*_*y*_ in the same way. Lastly, player *x* attempts to adopt the strategy *s*_*y*_ from player *y* with a probability determined by the Fermi function

$$W\left(s_{y}\to s_{x}\right)=\frac{1}{1+exp[\left(U_{x}-U_{y}\right)/K]},$$ (1)

where *K* = 0.1 quantifies the uncertainty related to the strategy adoption process [71]. During one full Monte Carlo step, each player on both networks has a chance to adopt one of the neighboring strategies once on average.

To incorporate individual heterogeneity into the coupling strength *α*, we can express the coupling strength *α* as follows

α=A*χ, (2)

where *χ* denotes a uniformly distributed number in the interval [−1,1], and $\int_{-1}^{1}A\chi d\chi =0$ ensures that the average value of coupling strength across both networks is zero. The tunable parameter *A* thus dictates the amplitude of coupling strength. Obviously, *A* = 0 decouples both networks and renders the traditional case to be recovered. The larger the value of *A* is, the stronger the heterogeneity of interdependency is. To mimick the realistic cases, Eq (2) can be divided into two cases as illustrated: the first case is the symmetric coupling (case **I**) that means the coupling strength between player *x* and its partner *x*′ is the same (*i.e*., *α*_*x*_ = *α*_*x*′_); another one is the asymmetric coupling (case **II**), which is denoted as *α*_*x*_ ≠ *α*_*x*′_ (namely, the random number *χ* is different for *x* and *x*′).

The results of Monte Carlo simulations presented below were obtained from *L* = 200 to 400 lattices. The key quantity, the fraction of cooperators **F*_*C*_*, was determined within the last 10^4^ full MCS steps after sufficient long relaxation time steps are discarded. If not stated, the total number of MCS steps is assumed to MCS = 5 × 10^4^. Moreover, since the heterogeneous coupling may introduce additional disturbances, the final results were averaged over up to 20 independent realizations for each set of parameter values in order to assure suitable accuracy.

## Simulation Results

**Fig 1:**
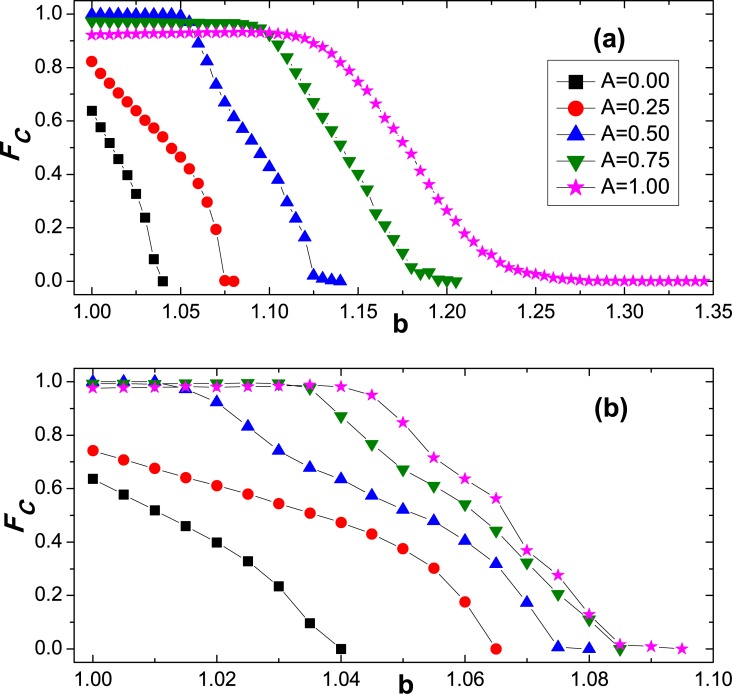
Stationary fraction of cooperators in the whole population (****F*_*C*_*** **) as a function of defection parameter (** ***b*** **) under different amplitude of coupling strength (** ***A***). On the top panel (a), corresponding players on two networks always have the same coupling strength which is taken from the interval [−*A*, *A*] according to Eq (2), and the cooperation can be greatly promoted as *A* increases; On the bottom panel (b), corresponding players on two networks hold distinct coupling strengths which are independently derived from Eq (2), and the cooperation is also promoted but the enhancement level is much smaller than that in the top panel. Other parameters are set to be *L* = 200 and *K* = 0.1.

To begin with, we examine how cooperation varies under the heterogeneous interdependency or coupling strength *A*. Fig 1 depicts the fraction of cooperators (**F*_*C*_*) as a dependence on *b* for different values of *A*. Compared with the traditional uncoupled case (namely, *A* = 0), it is clear that the increment of *A* will totally promote cooperation, irrespective of symmetric or asymmetric coupling. Moreover, the critical value *b*_*C*_, indicating the disappearance of cooperation, also enhances with *A* increasing. Since the larger *A* denotes stronger interdependence heterogeneity between networks, the observations suggest the consideration of both heterogeneous coupling promotes the evolution of cooperation, which further extends the content of interdependent network reciprocity [64].

Except for the joint advancement of cooperation level, we can also capture some difference for both heterogeneous coupling schemes. Compared with the case **II**, it is explicit that the elevation effect of case **I** is more pronounced, which means that cooperation becomes more robust and cooperators can resist the exploitation of defectors better. Under case **I**, the coupling strength between corresponding partners is symmetric, and the symmetric utility can easily render the corresponding agents to attain the consensus strategy. On the contrary, in case **II**, the interdependency values between each pair of players are independently taken from the uniform interval [-A, A], and the utility will be asymmetrically calculated and it is difficult to arrive at the coordination state.

**Fig 2:**
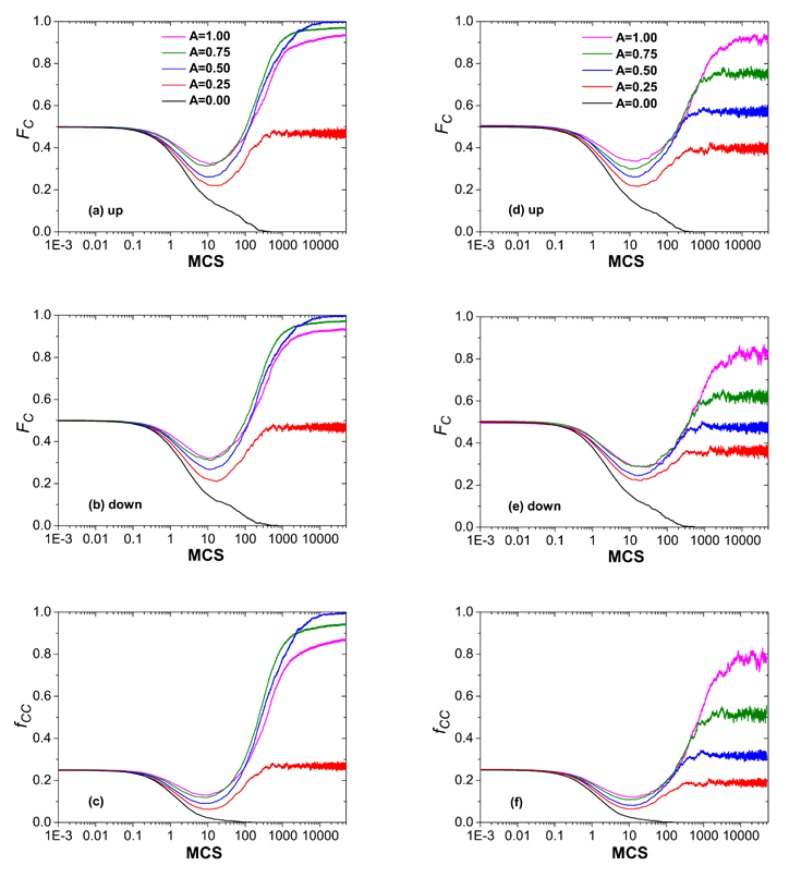
Time evolution of fraction of cooperators and cooperative pairs between two interdependent networks. The left three panels (a, b, c) depict the time course of evolution under the case **I**, where the corresponding players hold the same interdependency taken from the interval [−1,1] (i.e., *A* = 1) according to Eq (2). Panel (a) and (b) denote the fraction of cooperators on the upper lattice and lower one, respectively, and panel (c) represents the fraction of cooperative pairs which means the corresponding players are both cooperators on these two lattices. While for the case **II** in which each individual takes the interdependency value between −1 and 1, the right three panels, from panel (d) to (f), describe the time evolution of corresponding quantities. The defection parameter *b* is fixed to be *b* = 1.05, other parameters are set to be *L* = 200 and *K* = 0.1.

To further understand the origin of cooperation created by this type of interdependency, we explore the dynamical evolution of fraction of cooperation on two interdependent networks. Fig 2 presents the time course of cooperation fraction (**F*_*C*_*) and the percentage of cooperative pairs (*f*_*CC*_). Here, the cooperative pair (CC) means that a player at one lattice is a cooperator and the corresponding partner on the other lattice is also a cooperator. Similarly, we can define the cooperator-defector pair (CD, it means that a player on one lattice is a cooperator and corresponding partner is a defector on the other lattice) and defector-defector pair (DD, it points out that two matching players are both defectors on these two networks). Among them, the left three panels [from Fig 2a–2c] characterize the evolution of *F_C_* and *f*_*CC*_ under case **I**. We can clearly observe that, on one hand, the dynamical evolution of fraction of cooperators (*F_C_*) for two lattices can almost keep strictly consistent at each MCS step since the utility calculation of an individual equally considers the contribution from the payoff of corresponding partner; on the other hand, the evolutionary trend of **F*_*C*_* is qualitatively in step with that of *f*_*CC*_. In fact, we can also record the evolution of fraction of other strategy pairs (i.e., *f*_*CD*_ or *f*_*DD*_) during the Monte Carlo simulation (although *f*_*CD*_ and *f*_*DD*_ are not shown here), and find that only the evolution of *f*_*CC*_ dominates the cooperative behaviors on these two interdependent networks. In addition, there is also a subtle phenomenon to be noted that an optimal amplitude *A* exists here for the smaller defection parameter *b* = 1.05, and *A* = 0.5 leads to the highest fraction of cooperators which well agrees with the result in Fig 1.

At the same time, the right three panels [from Fig 2d–2f] illustrate the dynamical evolution of cooperation under the case **II**. Here, any pair of partners on two lattices will asymmetrically integrate the payoff from the opposite one into the fitness computation of focal player, and the fraction of cooperators (**F*_*C*_*) cannot completely fall into step with each other. However, the qualitative tendency is always kept to be consistent for **F*_*C*_* on two lattices, and thus the total fraction of cooperators will be obtained by averaging the fraction of cooperators on these two lattices as described in Fig 1. Again, it is also shown that the dynamical evolution of *F_C_* can qualitatively hold the same behavior as the fraction of cooperative pairs *f*_*CC*_. These results indicate that the evolution of cooperation between these two interdependent lattices is dictated by the CC strategy pairs.

**Fig 3:**
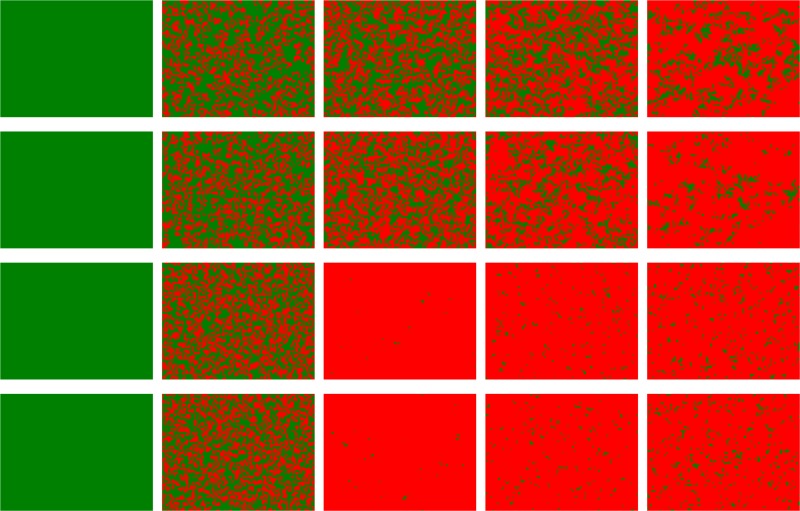
Characteristic patterns between cooperators and defectors on two interdependent lattices at MCS = 50000 when *A* is changed from 0 to 1.0. The panels for the first two rows denote the distribution of cooperators and defectors for the upper lattice and lower lattice in the case **II**; while the bottom two rows of panels represent the evolutionary patterns of players for the case **I**. In all these panels, red dots stand for cooperators and green dots represent defectors. From these figures, it is clearly shown that the fraction of cooperators is largely enhanced as *A* increases, meanwhile the cooperative pairs (C-C type coupling way) have the predominant advantage and dominate the evolutionary behavior on interdependent lattices. In particular, the designating way of the coupling strength between players on interdependent lattices can also affect the evolution of cooperation. The simulation parameters are set as follows: MCS = 50000, *b* = 1.05, *L* = 200 and *K* = 0.1, and *A* is varied from 0 to 1.0 with the step length 0.25 (from the left panels to the right ones).

To illustrate the evolution of cooperation in depth, we depict the characteristic snapshots of cooperators and defectors for various scenarios after enough time steps in Fig 3. In this figure, the first two rows of panels characterize the distribution of cooperators and defectors on two layered lattices, in which the coupling strength is independently set among players within each lattice according to Eq (2), that is, case **II**. Under this case, when *A* = 0, the system is reduced into two traditional lattices, all players cannot resist the temptation of defection and evolve into the full defection state on these two lattices. However, when *A* > 0, the evolutionary state can have a chance to escape from the fate of full defection and cooperators can organize into some cooperative clusters to avoid the employment of defectors. Thus, it is indicated that integrating the payoff from the corresponding player on the other lattice into the focal player’s fitness evaluation helps to promote the cooperation. Meanwhile, the clusters become larger and larger as *A* increases, and even a strongly connected giant component is created so that only the sporadic defectors exist in the upper and lower lattices when *A* = 1.0. Likely, the bottom two lines of panels describe the evolutionary patterns where the coupling strength is set to be same for corresponding players on two interdependent lattices, that is, case **I**. It is explicitly observed that the cooperation can be further elevated when compared to the case **II**, and an interesting phenomenon different from the case **II** is that the optimal *A* exists when *A* > 0 varies, and the current result is again consistent with the above-mentioned results. In the same manner, *A* = 0 leads to two independent traditional lattices.

**Fig 4:**
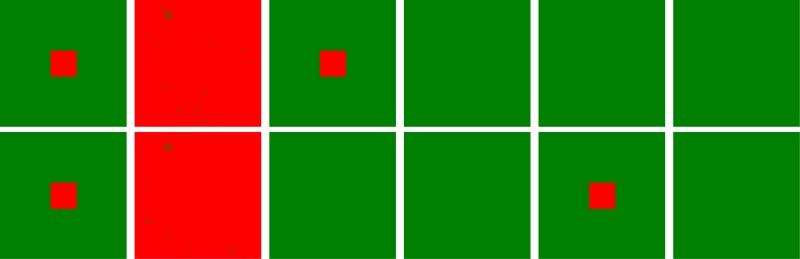
For a specific initial setup, characteristic patterns between cooperators and defectors on two interdependent lattices at MCS = 1 and MCS = 50000 under case I. In all panels, red dots represent cooperators and green dot denote defectors. For the left four panels, central cooperators are initially set on upper and lower lattices at the same time; only cooperators exist on the center of upper lattices in the middle four panels; while cooperators merely exist on the center of lower lattices in the right four panels. However, the coupling strength for each pair of players on two lattices is set to be equal, in which the coupling strength is taken from Eq (2). It is clearly indicated that cooperative pairs support the emergence of cooperation on interdependent networks. From left to right, the only difference lies that initial conditions are set as different parameter deployments between cooperators and defectors. The simulation parameters are set as follows: MCS = 50000, *b* = 1.05, *L* = 200, *K* = 0.1 and *A* = 0.5.

**Fig 5:**
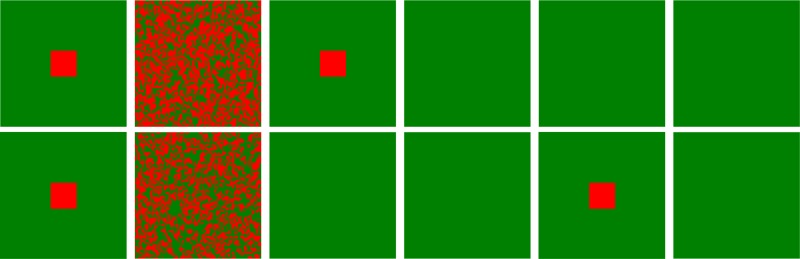
For a specific initial setup, the characteristic patterns between cooperators and defectors on two interdependent lattices at MCS = 1 and MCS = 50000 under case II. In all panels, red dots represent cooperators and green dot denote defectors. For the left four panels, central cooperators are initially set on upper and lower lattices at the same time; only cooperators exist on the center of upper lattices in the middle four panels; while only cooperators exist on the center of lower lattices in the right four panels. Here, the coupling strength for each player is independently assigned from Eq (2). Likewise, the coupling of C-C type setup facilitate the evolution of cooperation on interdependent networks, and only that the fraction of cooperators is slightly less than that under the **case I**. The simulation parameters are still set to be: MCS = 50000, *b* = 1.05, *L* = 200, *K* = 0.1 and *A* = 0.5.

In order to highlight the role of cooperative pairs (CC) in the evolution of cooperation on interdependent networks, we delineate the characteristic patterns at the last time step for different initial distribution of cooperators and defectors in Figs 4 and 5. Unlike the traditional setup, each player can take the **C** or **D** strategy with the identical possibility, here 40 × 40 players at the heart areas are cooperators at the upper and/or lower lattice and the rest ones are defectors. In Fig 4, the corresponding players between two lattices hold the same coupling strength mutually, and we can observe that the cooperation can persist only if the players in the central areas are both set to be cooperators on these two lattices, as shown in the left four panels. While for the middle or right four panels, it can be seen that the defectors will take over the whole system when only central players at the upper or lower lattice are set to be cooperators. Thus, the coupling between cooperators on these two interdependent lattices is the key to the evolution of cooperation. Similar phenomena can be identified in Fig 5 where corresponding agents independently take the value of coupling strength from Eq (2), and again validates the fact that the cooperative pairs (CC pairs) facilitate the cooperation behavior. The only difference is that the cooperation level under this case is much lower that that in case **I**.

**Fig 6:**
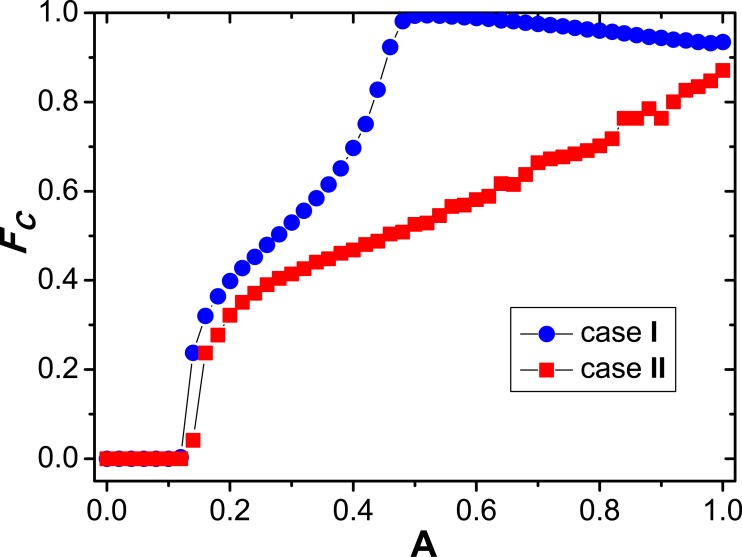
Relationship between the fraction of cooperators and the amplitude of coupling strength at a fixed defection parameter. In the whole, the tendency is that the cooperation will be elevated as *A* increases. However, under the symmetric case (*i.e*., case **I**), the optimal amplitude of coupling strength exists, but the asymmetric scenario (case **II**) leads to the monotonic variation of cooperation as coupling strength grows. The parameter setup is still set to be: MCS = 50000, *b* = 1.05, *L* = 200 and *K* = 0.1.

Additionally, we investigate the effect of amplitude of coupling strength (*A*) on the cooperation at a fixed defection parameter. In Fig 6, the fraction of cooperators (**F*_*C*_*) is plotted as a function of *A* when *b* is set to be 1.05. It is indicated that the cooperation can be promoted if *A* is beyond a specific critical threshold (*A*_*c*_). In reality, the coupling strength will be very small and can only integrate a very low percentage of payoff of corresponding player into the fitness value if *A* is less than *A*_*c*_, at this time the cooperation cannot be promoted and the full defection is arrived at. However, when *A* transcends over *A*_*c*_, the cooperation level can be greatly elevated for two types of coupling schemes with increasing *A*. An interesting phenomenon is found that the highest fraction of cooperator emerges at *A* ≈ 0.5 under the case **I**, where the full cooperation is almost reached within the whole population. Nevertheless, **F*_*C*_* will continue to expand for the second coupling mechanism when *A* adds up to the maximum (**A* = 1*), that is, there is no optimal effect concerning the cooperation. It is also worthy to be remarked that at any given value of *A*, the level of cooperation for the first fitness evaluation method (case **I**) will always be higher than that under the second scheme (case **II**). For this reason, the corresponding players on two interdependent lattices own the same coupling strength and resulting cooperative pairs with large coupling values may exist, which will greatly facilitate the evolution of cooperation. The current simulation again points out the origin of cooperation on the interdependent circumstances, and adding the payoff from the corresponding one on the other lattice into the utility estimation for the focal player on the one lattice is conducive to shedding some lights on the emergence and persistence of cooperation.

## Conclusions and Discussions

To summarize, the impact of network interdependency, which is quantified and characterized by the coupling between the payoff of corresponding partners on two interdependent lattices, on the evolution of cooperation in the spatial prisoner’s dilemma game is studied here in detail. In the current setup, we calculate an individual fitness by combing his/her own payoff on one lattice and the payoff from the corresponding partner on the other one, that is, the players will be virtually interrelated with or dependent on each other through the evaluation of fitness. However, the coupling strength is not a constant among player within the whole population, which is often assumed for most works regarding interdependent networked game at present, but uniformly taken from a specific interval [−*A*, *A*] according to Eq (2). Furthermore, we consider two different coupling schemes for the players on these two interdependent lattices. The first case is that the corresponding players on two lattices are hypothesized to hold the equal coupling strength (case **I**—the symmetric case) which is a random value between −*A* and *A*; In the second case, each player in the system independently and stochastically takes the value based on Eq (2), that is, the corresponding partners on two lattices may hold a completely different coupling strength (case **II**—the asymmetric case). Extensive simulations indicate that the interdependency or the coupling between players on two lattices can largely enhance the cooperation level within the whole population. Meanwhile, characteristic snapshots and cluster analysis point out that the cooperative pairs between corresponding partners on two lattices drives the evolution of cooperation. In addition, the symmetric coupling strength setup (case **I**) leads to the higher cooperation when compared to the asymmetric (case **II**), that is, the asymmetric coupling loses the evolutionary advantage. Our results convincingly demonstrated that the emergence or persistence of cooperation within many real-world systems can be accounted for by the interdependency between meta-populations or sub-systems, which deserves to be deeply explored in the future.
